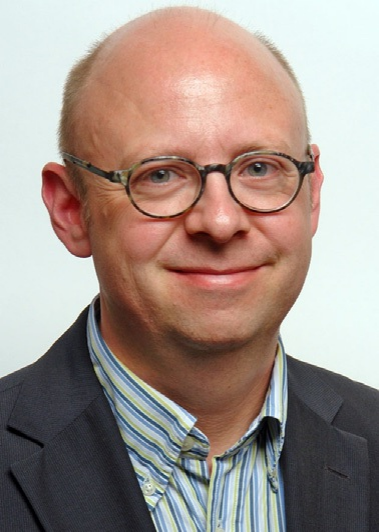# Structural Motifs of Alkali Metal Superbases in Non‐coordinating Solvents

**DOI:** 10.1002/chem.202002812

**Published:** 2020-11-09

**Authors:** Jan Klett

**Affiliations:** ^1^ Institut für Anorganische Chemie und Analytische Chemie Johannes Gutenberg-Universität Mainz Duesbergweg 10–14 55128 Mainz Germany

**Keywords:** aggregation, alkali metals, lithium, potassium, superbase

## Abstract

Lochmann–Schlosser superbases (LSB) are a standard reagent in synthetic chemistry to achieve an exchange of a proton on an organic framework with an alkali metal cation, which in turn can be replaced by a wide range of electrophilic groups. In standard examples, the deprotonating reagent consists of an equimolar mixture of *n*‐butyllithium and potassium *t*‐butoxide. However, the nature of the reactive species could not be pinned down either for this composition or for similar mixtures with comparable high reactivity. Despite the poor solubility and the fierce reactivity, some insights into this mixture were achieved by some indirect results, comparison with chemically related systems, or skillful deductions. Recent results, mainly based on new soluble compounds, delivered structural evidence. These new insights lead to advanced and more detailed conclusions about the interplay of the involved components.

## Graphical abstract

**Navigating** in treacherous but treasure‐laden seas where alkyllithium and potassium alkoxides were discovered by Lochmann and Schlosser mix. Little is known about the uncharted areas away from the well‐known coastal regions of the starting materials. Many molecular vessels were precipitated and lost here. Progress was finally made by careful hopping from molecular island to island.

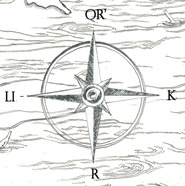

## The Organometallic Chemistry of Alkali Metals

The organometallic chemistry in general, and the organometallic chemistry of the alkali metals in particular, is based on the seemingly unfavorable bond between the carbon atom of an organic group and the metal atom. At first glance, this arises from the tendency of carbon to form covalent bonds, while many metals prefer more ionic interactions. Schlosser considered this explanation as being too simple.^[1]^ He points out the highly polar character of the metal–carbon bond. This polarity is based on the difference of the corresponding electro‐negativities, leading to a negatively polarized carbon as evidenced by experimental facts.^[2]^ Schlosser emphasizes that the situation with the alkali metal atom should not be neglected; even with the polar bond, the metal atom is not keen to go without further bonded electrons. Yet, a single, weak bond cannot serve the electronic needs of the metal atom. The solution is in the formation of multiple weak bonds. The metal atom is flexible in its coordination sphere, both in the number and the geometric arrangement of ligands. This is not the case for the carbon of an organic group attached to the metal atom. Due to the covalently attached atoms on the carbon atom (e.g. carbon or silicon centered groups), its coordination to metal atoms is limited in terms of direction.

In other words, the negative charge of these organic groups is spatially directed towards one, two, or three metal atoms. This electronic situation leads to the formation of oligomers or larger aggregates, which can be derived as sections from salt structures, which was aptly demonstrated in a review by Stalke et. al.^[3]^ Both effects, the metal–carbon bond polarization and the tendency to form oligomers, play important roles with respect to the reactivity of organometallic alkali metal compounds or their interaction with other molecules. The negative charge on the carbon atom renders it a very strong reducing agent on one side, as well as a reactive Lewis base, leading to nucleophilic behavior or high Brønsted basicity on the other. The degree of aggregation of organometallic alkali metal compounds has a large influence on the solubility of such compounds; quantitative effects on the reactivity are also discussed.^[4]^ However, the influence of different grades of aggregation on reaction rates is far from being simple.^[5]^

At this point, it is very important to mention the necessary distinction between different classes of organic groups in organometallic compounds. The sp^3^‐hybridized carbon atom of an aliphatic alkyl group bonded to the metal shows low group electronegativity, and acts as a donor with the hapticity η^1^; nevertheless, it can adopt bridging positions to metals. sp^2^‐ and sp‐hybridized carbon atoms of allyl, aryl, or ethynyl groups show higher electronegativity due to the greater s‐character of the involved orbitals. In addition, they can accept further, bridging interactions to metals using π‐orbitals.^[2b]^ This greatly affects the reactivity and aggregation of such compounds. In the following, the focus is set on the organometallic chemistry of alkyl groups. This includes their high basicity and their characteristic modes of interaction with alkali metals in solid state and in solutions of non‐coordinating solvents.^[6]^

## Organometallic Compounds of Lithium and the Heavier Alkali Metals

**Scheme 1:**
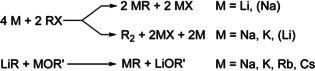
Direct synthesis of alkali metal alkyl compounds with the alternative Wurtz–Fittig “side‐reaction”, and the metal‐metal exchange reaction producing alkyl compounds of the heavier alkali metals (X=halogen, R=alkyl, OR=alkoxy).

Organolithium compounds are dominating organometallic chemistry, while their heavier alkali metal congeners play only a minor role. At first glance, the straight access to organolithium compounds^[7]^ by direct synthesis (reaction of lithium metal with organic halide) is a substantial advantage (Scheme 1).

Some examples of this method are reported for heavier alkali metals,^[8]^ but only few compounds were characterized.^[9]^ This approach is more susceptible to a Wurtz–Fittig reaction during synthesis,^[10]^ leading to low or negligible yields. The corresponding lithium compounds are more robust to this specific side‐reaction,^[11]^ which allows their isolation with good yields and high purity. However, the organometallic chemistry of the heavier alkali metals^[12]^ with their highly ionic metal–carbon interaction is further complicated by two other fundamental characteristics: aggregation and reactivity. The ionic interactions and the larger size of the metal atoms lead to high coordination numbers and polymeric aggregates, resulting in negligible solubility.^[13]^ Coordinating solvents such as benzene, toluene, or ethers,^[14]^ which could dissolve these compounds and facilitate their characterization and synthetic use, suffer chemical attacks caused by the inherent reactivity.^[15]^ Another route of decomposition is β‐elimination, which affects alkyllithium compounds^[16]^ and becomes even more destructive for the heavier alkali metal alkyl congeners.^[17]^ Despite these problems, it is attractive to harvest this formidable reactivity in hydrogen‐metal exchange/deprotonation/metalation reactions, which cannot be achieved by corresponding lithium compounds.

## Access to Highly Reactive Alkali Metal Bases

**Scheme 2:**
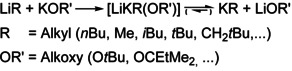
Formation of alkylpotassium (and lithium alkoxide) by a reaction of alkyllithium and potassium alkoxide. The essential but unknown intermediate is often referred to as Lochmann–Schlosser superbase.

Two chemically possible options can be considered to achieve this goal: Either the organometallic compounds of the heavier alkali metals are brought into a more controllable and synthetically usable form, or the reactivity of organolithium compounds is increased significantly without sacrificing their already positive and very useful characteristics.^[18]^ Schlosser described how this was achieved by the search for activating ligands for organolithium compounds.^[19]^ Ethers,^[14]^ (including crown ethers) and (chelating) tertiary amines,^[20]^ are able to cause this activation of organolithium, but suffer metalation themselves under these conditions. Chelating di‐alkoxides offer the Lewis donor capabilities and chemical inertness, but lack the necessary solubility in hydrocarbon solvents such as *n*‐hexane. The only remaining possibility is the use of tertiary alkoxides with feasible solubility, such as lithium, sodium, or potassium *tert*‐butoxides. The combination of alkyllithium with lithium alkoxides produces compounds,^[21]^ the reactivity of which (in addition‐reactions)^[22]^ does not substantially differ from the alkyllithium itself.^[23]^ Potassium *tert*‐butoxide as “ligand” in mixtures with alkyllithium ultimately leads to the desired increased reactivity (Scheme 2). Exactly the same reaction is used to produce alkylpotassium from the corresponding lithium compound. This metal‐metal exchange reaction had already been investigated in depth by Lochmann and his group in the mid‐sixties.^[24]^ The positive effect of alkoxide on metalation reactions of alkenes with amylsodium (*n*‐pentylsodium) had already been studied by Morton two decades earlier.^[25]^ Here, the sodium alkoxide was formed in situ by the sacrificial reaction of a part of the amylsodium with *iso*‐propanol. Due to the limited access to amylsodium, this line of research was not developed further. The majority of examples refers to *n‐*butyllithium, but examples using methyllithium,^[26]^ *iso*‐butyllithium,^[27]^ or *tert*‐butyllithium^[28]^ are also reported.

A number of excellent reviews have been published by Lochmann,[^23^, ^29^] Schlosser,[^15^, ^30^] and others,^[31]^ discussing the nature of these superbases, their reactivity towards organic substrates, and changes in the composition by variations of alkyl/alkoxy ratios. A number of studies with more or less related systems were aimed at finding out the chemical nature of these superbases. A publication by Bauer and Lochmann provides an excellent overview of experiments conducted until then.^[32]^ However, the majority of conclusions are drawn from products, selectivities, and yields of the reaction of corresponding basic systems with organic substrates and not from the LSBs themselves. According to the interaction of the base components with each other, the results are often inconclusive or contradictory. Nevertheless, the review^[29]^ by Lochmann summarizes some important conclusions: The reaction of alkyllithium with potassium alkoxides produces alkylpotassium, the products of subsequent reactions also give rise to corresponding potassium compounds. The potassium alkoxide has to be used in at least equimolar compounds, so its role cannot be described as merely catalytic or activating; the contribution of the participating lithium alkoxide is less dominant. The reactivity benefits from the use of potassium alkoxides bearing more branched groups to increase solubility and concentration. In summary, these mixtures are highly flexible systems, in which the metal‐metal interchange plays an “integral part in the reaction”.

## Alkali Metal Alkoxides

In the vast majority of examples, the alkali metal alkoxides used in LSBs are potassium compounds (LiR/KOR’). Investigations also included mixtures of alkyllithium with sodium alkoxides^[33]^ (LiR/NaOR’) or combinations of alkyl sodium with sodium alkoxide^[34]^ (NaR/NaOR’) or potassium alkoxide^[35]^ (NaR/KOR’). Very few examples involve rubidium or cesium alkoxides^[36]^ (LiR/RbOR’ and LiR/CsOR’). Secondary alkoxides^[25]^ or bifunctional alkoxides (such as pinacolate)^[35]^ are rarely used. This can be attributed to low solubility or a lack of chemical inertness. Most studies cover reactions with tertiary alkoxides. In some cases, the use of branched tertiary alkoxide (such as 2‐methyl‐2‐butoxide or 3‐methyl‐3‐pentoxide) instead of *tert*‐butoxide led to better results. Therefore, the mixtures featuring branched alkoxides with improved solubility are an advancement and hence called *LSBs of the second generation*.^[23]^

**Figure 1:**
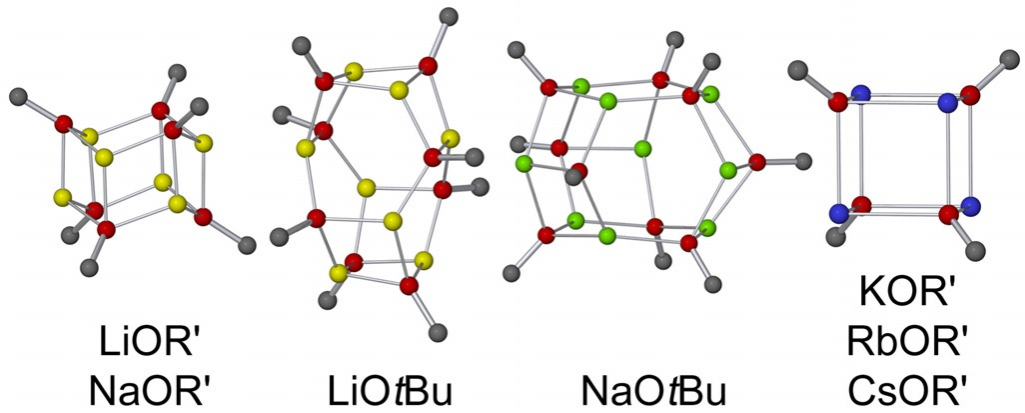
Oligomeric structures of alkali metal alkoxides. Only the tertiary carbon atoms are shown; the adjacent alkyl groups are omitted for clarity. Li, yellow; Na, green; K, Rb, or Cs, blue; O, red; C, dark grey.

Structural motifs found in solid‐state structures of relevant alkali metal alkoxides (Figure 1) might be reflected in the structures of corresponding mixed aggregates.^[37]^ In tertiary alkoxides, every alkali metal atom interacts with three oxygen atoms of three alkoxy groups, and vice versa. This always results in the formation of M_2_O_2_ four‐membered rings; also M_3_O_3_ six‐membered rings are possible.^[38]^ LiO*t*Bu is found in hexameric aggregates,^[39]^ but also octameric aggregates^[40]^ are observed. NaO*t*Bu crystallizes as hexamer and nonamer side by side.^[39]^ The corresponding potassium, rubidium, and cesium *tert*‐butoxides form regular heterocubanes.^[41]^ The structures of alkali metal 2‐methyl‐2‐butoxides (*tert*‐amyloxide) follow this pattern but show increased solubility.^[42]^

The reason for the limitation to tetrameric structures in the case of potassium, rubidium, and cesium originates from the possibility to create mutual intermolecular interactions, which can be optimally arranged in a packing of tetramers. Tetramers are also found in lithium^[43]^ and sodium^[44]^ alkoxides, if more bulky alkoxy groups such as OCH(*t*Bu)_2_ or OC(CF_3_)_3_ are used. The co‐existence of hexamers and octamers (in the case of LiO*t*Bu) provides a clue, how smaller units such as dimers are transferred between oligomers and mixed alkyl/alkoxy aggregates.

## Working around the Chemistry of Alkali Metal Superbases

**Scheme 3:**
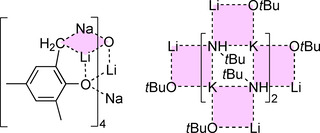
Schematic representations of the mixed aggregate compounds by Harder and Streitwieser (left), and by Mulvey et al.(right). The relevant Li/K‐element arrangements are highlighted. Additional coordinating molecules (Na: TMEDA, K: benzene) were omitted for clarity.

Two important structural examples pointed in the direction of which structural motifs are to be expected in bi‐metallic and hetero‐anionic systems (Scheme 3). Harder and Streitwieser used the reaction of sodium phenoxide with *n*‐butyllithium to produce an intramolecular combination of lithium phenoxide with a sodium‐metalated benzyl‐position.^[45]^ This structurally characterized molecule combines a heavier alkali metal (sodium) carbon interaction with lithium oxygen interactions, two expectable arrangements in LSBs.

A step further is a result by Mulvey et. al., which consisted of the combination of a lithiated primary amine with potassium *tert*‐butoxide, which is able to perform a metalation of toluene.^[46]^ Here, the metal atoms (lithium/potassium) are combined with an alkoxide/amide framework; the amide anion is isoelectronic to the corresponding alkyl groups (e.g. in *n‐*butyllithium). However, both examples, though presenting relevant metal‐element interactions, also feature the possibility of additional Lewis‐base/metal interactions (π‐ or additional free electron pairs) combined with less Brønsted‐basic groups (benzylic M‐CH_2_Ph and M‐NR_2_ versus aliphatic M‐CR_3_).

**Scheme 4:**
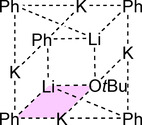
Schematic representation of the mixed aggregate compound reported by Strohmann. Only one of the three relevant metal‐element interactions is highlighted; additional coordinating THF and benzene molecules are omitted for clarity.

A new level was reached in the publication by Strohmann et al.:^[47]^ here, a structure was presented that incorporates a combination of phenyl lithium and phenyl potassium with lithium *tert*‐butoxide, with THF as additional donor (Scheme 4). Both groups of metals exhibit interactions with alkoxide oxygen atoms and phenyl carbon atoms at the same time.

Though the basicity towards toluene could be demonstrated and relevant structural goals were achieved, three more obstacles are evident in the way towards structurally identified Lochmann–Schlosser superbases: (i) The basicity of the phenyl group can be expected to be lower in comparison to the corresponding alkyl groups, based on the higher electronegativity of the phenyl carbon (vide supra). (ii) The phenyl groups can interact with metal cations both through the electron pair of the *ipso*‐carbon atom and as well through the π ‐system of the phenyl ring.^[2]^ This creates structural motifs, which cannot be present in alkyl systems. (iii) The use of THF adds a new component to the system that adds additional structural properties as well as proton‐acidic reactivity, which must be suppressed by low temperatures when used in Lochmann–Schlosser superbasic mixtures. However, the relevance of this example, which no longer represents a mere model system, cannot be underestimated with respect to aromatic reaction products resulting from superbasic metalations, or for the Lochmann–Schlosser superbase chemistry in THF at low temperatures.

**Scheme 5:**
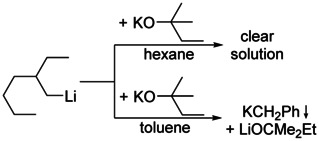
Reaction of 2‐ethylhexyllithium with potassium *tert*‐amyloxide and the reaction of this mixture with toluene.

What is the main obstacle on the way to achieve the isolation and characterization of compounds very similar (or even identical) to those expected to be present in LSBs? The main challenge is the extremely low solubility of the corresponding alkylpotassium, which is ultimately always formed in these superbasic mixtures (Scheme 2). This forces every equilibrium involving alkylpotassium to the product side, making it impossible to identify relevant compounds due to their very low concentration. An example of a highly soluble Lochmann–Schlosser superbase, which is formed in hexane by combining 2‐ethylhexyllithium with potassium *tert*‐amyloxide [KO*t*Am] (Scheme 5), is reported,^[48]^ however, without further data on the mixture itself. This might be caused by the low thermal stability of the formed 2‐ethylhexylpotassium, which is reported to be soluble in hexane.

**Scheme 6:**
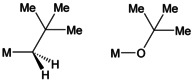
Structural similarity between neopentyl (left) and *tert*‐butoxide (right), M=alkali metal. The main differences lie in the spatial requirements of the two hydrogen atoms and the different M‐C−C and M‐*O*‐C angles.

In our own research, we found that mixtures of potassium *tert*‐ butoxide with neopentyllithium^[49]^ [LiCH_2_ *t*Bu, LiNp] also produced solid neopentylpotassium^[50]^ [KCH_2_ *t*Bu, KNp]. The precipitate was isolated by filtration. However, the yield of isolated KNp was considerably lower compared to the results of similar potassium compounds.^[51]^ In fact, it was possible to isolate a crystalline solid from the filtrate, which contained all four components expected to be presents in LSBs: Lithium, potassium, alkyl groups, and alkoxide groups.^[52]^ One reason for the unexpected high solubility of the neopentyl/alkoxide mixed aggregates is the structural similarity between *tert*‐butoxy groups [**O**‐*t*Bu] and Np groups [**CH_2_**‐*t*Bu] (Scheme 6). The structural mimicry of the Np group leads to a statistical replacement of O*t*Bu groups, leading to decreased symmetry of the resulting molecules, and in turn to an increased solubility.

## Possible Combinations in a Four‐Component System

In order to obtain a better overview of the available results, a general reflection of the possible methods to combine lithium and potassium atoms with alkoxy and alkyl groups in one compound might be helpful. Schlosser did not expect the formation of a single mixed aggregate, but an entire family of such adducts.^[19]^ In this respect, a systematic approach to which family members can be expected in such systems may produce additional insights.

The hypothetical combination of all four components, lithium, potassium, alkyl groups [R], and alkoxy groups [OR’], will result in the formation of a compound with the formula Li_*x*_K_*y*_R_*z*_(OR’)_*x*+*y*−*z*_. Bearing in mind that the single compounds LiR, KR, LiOR’, and KOR’ possess lower or at least different reactivity than mixed aggregates; the simplest relevant combination is formed by one equivalent LiR and KOR’ each: LiKR(OR’). This compound could also be seen as hetero‐dimer, with two metal atoms present: LiR⋅KOR’ (or LiOR’⋅KR). In reality, the number of metals will be two or higher. Examples from alkali metal alkoxide compounds suggest the formation of hetero‐tetramers, ‐hexamers, ‐octamers, or ‐nonamers. However, also hetero‐forms of trimers, pentamers, or heptamers are feasible. Hetero‐octamers are of particular interest, because two model compounds possess the composition of the mixed alkyl/alkoxy lithium compound Li_8_(*n*Bu)_4_(O*t*Bu)_4_ (or 4Li*n*Bu⋅4LiO*t*Bu),^[21]^ and the lithium/potassium alkoxide Li_4_K_4_(O*t*Bu)_8_ (or 4LiO*t*Bu⋅4KO*t*Bu).^[53]^ Both compounds may offer a possible structural design for aggregates combining all four components. The question is whether or not the introduction of alkyl groups or potassium atoms will have a more dominant effect on the resulting structure.

**Figure 2:**
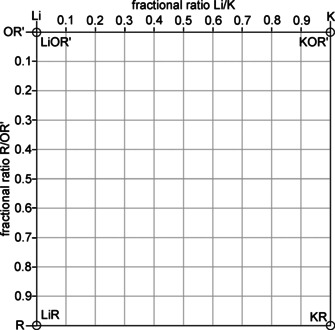
Graphical representation of the composition of mixed aggregates consisting of lithium and potassium atoms and alkyl and alkoxy groups as double binary phase diagram. Li compounds are found on the left, K compounds on the right edge; alkoxy compounds are situated at the top and alkyl compounds at the lower edge. The corresponding pure alkali metal compounds (alkoxy or alkyl) are found in the corners of the diagram (circles). The edges between the corners are populated by compounds where either the metals or the alkyl and alkoxy groups are mixed. Compounds in the area represent four component Li/K/alkyl/alkoxy mixed aggregates.

To obtain a clearer picture of the possible hetero‐oligomers, one should keep in mind that the number of cationic and anionic units must be the same. This obvious condition simplifies the formula Li_*x*_K_*y*_R_z_(OR’)_*x*+*y*−*z*_ with three variables to a system with only two fractional variables: Li_*a*_K_(1‐*a*)_R_*b*_(OR’)_(1−*b*)_. To avoid fractional numbers of atoms or groups, it is possible to add a variable m, which also reflects the degree of oligomerization: Li_*ma*_K_*m*(1−*a*)_R_m*b*_(OR’)_*m*(1−*b*)_. The simplification of a fractional Li/K ratio (a) and a fractional R/OR’ ratio (b) allows plotting all possible variations in a two dimensional coordination system referring to a and b (Figure 2).^[54]^

In the chosen representation, pure lithium compounds can be found on the left, pure potassium compounds on the right border. Pure alkoxides are found at the top of the diagram, alkyl compounds at the lower end.

**Figure 3:**
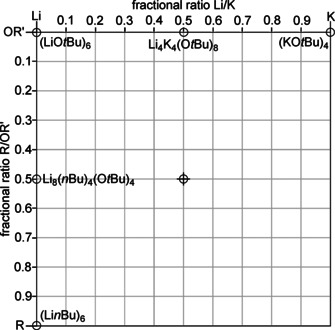
Same graphical representation as Figure 2 with representative compounds [(LiO*t*Bu)_6_, (KO*t*Bu)_4_, and (Li*n*Bu)_6_)] in the corners (circles). In the middle of the right and top edges are the compounds Li_8_(*n*Bu)_4_(O*t*Bu)_4_ ^[21]^ and Li_4_K_4_(O*t*Bu)_8_.^[53a]^ The molecular 1:1 mixed aggregate of *n‐*butyllithium and potassium *tert*‐butoxide represents an ideal LSB situated in the middle of the diagram (cross).

The fractional ratios shown on the diagram axes are defined by the number *n* of each component: Li/K: *n*(K)/[*n*(Li)+*n*(K)] and R/OR’: *n*(R)/[*n*(OR’)+*n*(R)]. Accordingly, the pure alkyl or alkoxy compounds can be found in the corners of the diagram: lithium alkoxide LiOR’ at the top left, potassium alkoxide KOR’ at the top right corner, alkyllithium LiR and alkylpotassium KR in the bottom left and bottom right corners, respectively. Every mixed aggregate consisting of three or more of these four components (Li, K, R, OR’), independent from its existence in solution or solid state, can be placed on the edges (three components) or the area (four components) of this diagram. This raw diagram can then be populated with substances relevant for this type of system (Figure 3): lithium *tert*‐butoxide LiO*t*Bu, which can be found both in hexameric^[39]^ [Li_6_(O*t*Bu)_6_] or octameric^[40]^ [Li_8_(O*t*Bu)_8_] form; tetrameric potassium *tert*‐butoxide [K_4_(O*t*Bu)_4_];^[41a]^ and hexameric butyllithium [Li_6_(*n*Bu)_6_].^[55]^ No relevant alkylpotassium compound is known, but examples of alkylsodium^[9c]^ or donor coordinated alkylpotassium^[51]^ hint towards the possible existence of tetrameric units [K_4_R_4_].

The two substances Li_8_(*n*Bu)_4_(O*t*Bu)_4_ ^[21]^ and Li_4_K_4_(O*t*Bu)_8_,^[53a]^ which act as potential structural models for Lochmann–Schlosser superbases, are found at the center of the respective edges of the diagram. The first compound has a structure similar to octameric [Li_8_(O*t*Bu)_8_], with the *n‐*butyl groups on the peripheral positions of the molecule. A Lochmann–Schlosser superbase, consisting of an ideal 1:1 combination of *n‐*butyllithium and potassium *tert*‐butoxide, would be situated in the middle of the diagram. The lack of compounds populating the diagram area can be attributed to the removal of alkyl potassium (right lower corner) from every conceivable mixed aggregate containing both potassium and alkyl groups. The very low solubility in most solvents removes it from the corresponding equilibria (Scheme 2). The remaining compounds without potassium or alkyl group are enriched with lithium alkoxide, which places them on the edges of the diagram. The mixed aggregate of the potassium‐rich compound reported by Strohmann^[47]^ (Scheme 4) could be seen as an exception. However, in this case, the strongly coordinating solvent THF plays a dominant role and this discussion is restricted to systems in the absence of donor solvents.

The same position of mixed aggregates with the same fractional composition of each component, but with different degrees of oligomerization (e.g. Li_2_K_2_R_2_(OR’)_2_, Li_3_K_3_R_3_(OR’)_3_, or Li_4_K_4_R_4_(OR’)_4_), is a drawback of this plot, but experimental results will show whether these combinations exist side by side.

## The Neopentyl Group as Key to Mixed Alkyl/Alkoxy Aggregates

After the preparation of the field, it is necessary to find a way to solubilize alkyl potassium to lift the restriction to the sideline. As described above, we managed this by using neopentyllithium instead of *n*‐butyllithium as alkyl source in LSB mixtures.^[52]^ The combination of structural similarity to the *tert*‐butoxy group and the introduction of structural disorder hindering crystallization and precipitation (vide supra) considerably increases the solubility of the corresponding compounds. An additional advantage of neopentyl and *tert*‐butoxy from a practical point of view is the simplicity of the resulting ^1^H NMR spectra. The presence of three resonances in ^1^H NMR (Np: CH_2_ and C(CH_3_)_3_; O*t*Bu: C(CH_3_)_3_) makes it easier to identify these groups even in structurally different species.

**Scheme 7:**
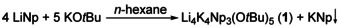
Reaction of LiNp and KO*t*Bu in *n*‐hexane resulting in the formation of soluble compound **1** and the precipitation of KNp.

In the attempt to produce neopentylpotassium [KNp] in a reaction using neopentyllithium and potassium *tert*‐butoxide (Scheme 2), we noticed the poor yield of KNp (<30 %). Considering the low solubility of pure samples of KNp in C_6_D_12_ for NMR spectroscopy, we realized that the excess KNp must be part of a soluble mixed aggregate. From the mother liquid of the reaction mixture, we obtained crystals at −30 °C with the approximate formula Li_4_K_4_Np_3_(O*t*Bu)_5_ (**1**, Scheme 7).

**Figure 4:**
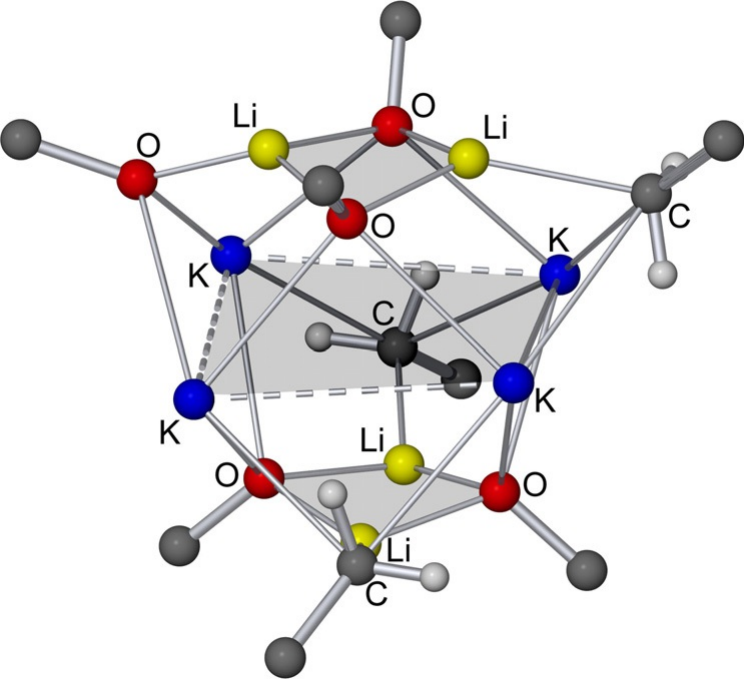
Molecular structure of compound **1**. Minor occupied disordered units and *tert*‐butoxy/neopentyl methyl groups are omitted for clarity. Li_2_O_2_‐ and K_4_‐squares are shaded for emphasis.

Compound **1** was characterized by ^1^H, ^13^C, and ^7^Li NMR spectroscopy as well as by X‐ray diffraction. The structure of **1** in solid state (Figure 4) was revealed as mixed Li/K/Np/O*t*Bu aggregate closely related to the mixed metal alkoxy compound reported by Mulvey,^[53a]^ Li_4_K_4_(O*t*Bu)_8_. This indicates that the co‐presence of lithium and potassium renders Li_4_K_4_(O*t*Bu)_8_ a more accurate model compound than Li_8_(*n*Bu)_4_(O*t*Bu)_4_ ^[21]^ in this case. Both Li_4_K_4_(O*t*Bu)_8_ and **1** show a planar square of four cationic potassium atoms, which is coordinated from both sides by two lithium alkoxide units (Figure 4). In the case of Li_4_K_4_(O*t*Bu)_8_, these two anionic units show the formula [(O*t*Bu)Li(O*t*Bu)_2_Li(O*t*Bu)]^2−^, while in **1**, the anionic units consist of a central Li_2_(O*t*Bu)_2_ dimer with statistical disordered terminal Np groups or O*t*Bu groups attached to both lithium atoms: [(Np/O*t*Bu)Li(O*t*Bu)_2_Li(Np/O*t*Bu)]^2−^_._ These anionic units are arranged in a staggered conformation, so that the terminal oxygen or carbon atoms form a tetrahedron with the potassium atoms approximately on four of the six edges.

The four *tert*‐butoxy groups in bridging positions between two lithium atoms with a μ_4_‐Li_2_K_2_ environment are not affected by the statistical disorder with neopentyl groups. Only the terminal groups of the [(Np/O*t*Bu)Li(O*t*Bu)_2_Li(Np/O*t*Bu)]^2−^ with a μ_4_‐Li_2_K environment show this replacement of *tert*‐butoxy groups by neopentyl groups. These neopentyl groups are also affected by a further positional disorder. In compound **1**, an average of ≈70 % of the four terminal *tert*‐butoxy groups are replaced by neopentyl compared to Li_4_K_4_(O*t*Bu)_8_, resulting in an approximate composition of Li_4_K_4_Np_2.75_(O*t*Bu)_5.25_.

## Behavior of Mixtures of Neopentyllithium and Potassium *tert*‐Butoxide in Solution

**Scheme 8:**
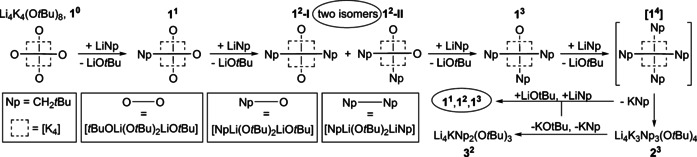
Step by step exchange of *tert*‐butoxy groups in Li_4_K_4_(O*t*Bu)_8_ (**1^0^**) with neopentyl groups, leading to the formation of neopentyl‐enriched compounds **1^1^**, **1^2^**, and **1^3^** (the superscripted number describes the number of alkyl groups in the mixed aggregate). Further introduction of neopentyl groups leads to the formation of the lithium‐rich compounds **2^3^** and **3^2^** before reaching the ideal combination of hypothetical **1^4^**.

The disordered and fractional composition of **1** and its close relationship with Mulvey's mixed alkoxy compound Li_4_K_4_(O*t*Bu)_8_ (by analogy compound **1** with no neopentyl group: **1^0^**) opened up interesting possibilities. Disorder between two chemically different groups is not desirable in terms of structural accuracy. However here, the structure of **1** represents two or more members of the same structural family with different numbers of neopentyl groups at the same time. **1** can be seen as a presentation of different orientations of **1^3^** but, at the same time, it also represents the compounds **1^2^** and potentially **1^4^** with a missing or an additional neopentyl group, respectively. By adding increasing amounts of LiNp to Li_4_K_4_(O*t*Bu)_8,_ **1^0^** (continuous variation^[56]^), it was possible to study its transformation into **1** by ^1^H NMR spectroscopy.^[52]^ LiNp was added to equimolar mixtures of LiO*t*Bu and KO*t*Bu in *n*‐hexane in increasing amounts; the crystals obtained at −30 °C were characterized by ^1^H NMR spectroscopy in deuterated cyclohexane [C_6_D_12_]. The results show that the intensity of one of the two distinguishable *t*BuO signals of Li_4_K_4_(O*t*Bu)_8_ is reduced while both singlet resonances of corresponding neopentyl groups (CH_2_ and *t*Bu) increase in intensity. This observation suggests the presence of Li_4_K_4_Np(O*t*Bu)_7_ (**1^1^** with one alkyl group) in solution with the arrangement similar to Li_4_K_4_(O*t*Bu)_8_ (**1^0^**), as was anticipated based on the group disorder (Np/O*t*Bu) present in the structure of **1**. Accordingly, this replacement of O*t*Bu by Np can be expected to occur exclusively in the peripheral position of a [(O*t*Bu)Li(O*t*Bu)_2_Li(O*t*Bu)]^2−^ unit (Scheme 8). Further addition of Np leads to the formation of Li_4_K_4_Np_2_(O*t*Bu)_6_ (**1^2^**). However, the addition of the second neopentyl group leads to the formation of two isomers: introduction of a second Np in the same Li(O*t*Bu)_2_Li centered unit together with the first Np group (**1^2^‐I**), or it can be placed next to the Li(O*t*Bu)_2_Li unit on the other side of the K_4_ plane (**1^2^‐II**). While **1^2^‐I** exhibits a symmetric arrangement, in **1^2^‐II**, the two protons of the CH_2_‐Np group have a different chemical environment, regardless of their rotational orientation.

**Figure 5:**
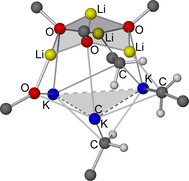
Molecular structure of compound **2**. Minor occupied disordered units and *tert*‐butoxy/neopentyl methyl groups are omitted for clarity. Li_2_O_2_‐squares and the K_3_‐triangle are shaded for emphasis.

**Figure 6:**
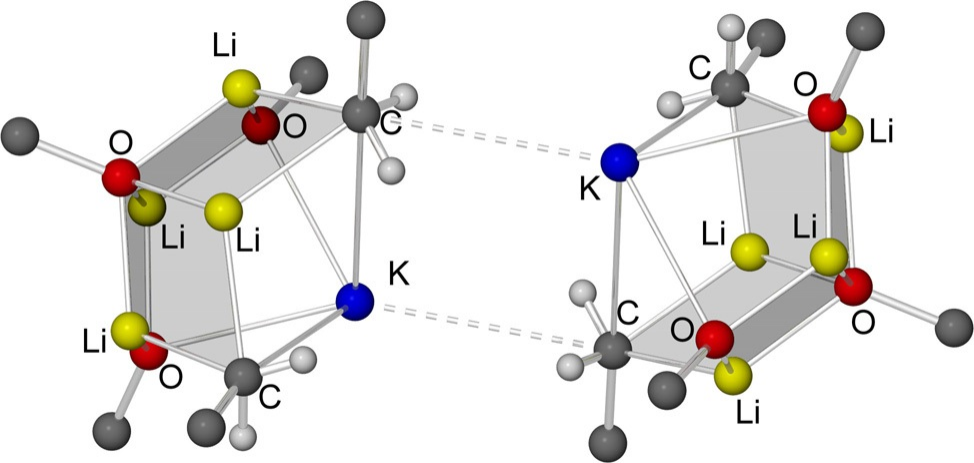
Molecular structure of dimeric compound **3**. Minor occupied disordered units and *tert*‐butoxy/neopentyl methyl groups are omitted for clarity. Li_2_O_2_‐squares are shaded for emphasis.

This break in symmetry manifests itself in the ^1^H NMR spectrum by the diastereotopic splitting of the CH_2_ signal into two symmetric duplets. In the ^1^H NMR spectrum, **1^2^‐I** just shows a singlet replacing or adding to the singlet of **1^1^**. These findings support the existence of **1^1^**, **1^2^‐I**, and **1^2^‐II** in solution at room temperature. When even more LiNp is used in preparation of the crystalline samples, the corresponding ^1^H NMR spectra become more complicated. For Li_4_K_4_Np_3_(O*t*Bu)_5_ (**1^3^≈1**) in C_6_D_12_, the expected signals in the CH_2_‐Np region are found (singlet + two diastereotopic duplets), but a new broad signal appears also. This shows that in solution compound **1**, which was isolated as pure crystalline solid, partially falls apart into other species. In such solutions the outcome of crystallization depends on the concentration and the solubility of the compounds formed in the solution equilibrium at a given temperature. The replacement of more than two or three of the peripheral O*t*Bu groups in Li_4_K_4_(O*t*Bu)_8_ leads to a structural instability due to weaker metal‐Np interactions. Accordingly, even higher Np contents did not lead to the formation of the “ideal” neopentyl LSB, but to the formation of mixed aggregates enriched with lithium and alkyl/alkoxy ratios close to 1/1. The two compounds Li_4_K_3_Np_3_(O*t*Bu)_4_, **2** (≈**2^3^**) (Figure 5) and Li_4_KNp_2_(O*t*Bu)_3_, **3** (≈**3^2^**) (Figure 6) were isolated from such mixtures and characterized by X‐ray crystallography and NMR spectroscopy. Again, both compounds exhibit partial group disorder between O*t*Bu and Np as seen before in compound **1**, suggesting the existence of neopentyl‐rich compounds such as Li_4_K_3_Np_4_(O*t*Bu)_3_, **2^4^** and Li_4_KNp_3_(O*t*Bu)_2_, **3^3^**.

The structure of **3** shows the formation of dimers. Additional intermolecular interactions are found between the potassium atom and the CH_2_ unit of the neighboring Np group. The two protons are orientated towards the potassium in an agostic type interaction. This weaker long‐range interaction is possible because both the potassium atoms and the neopentyl groups are found in exposed positions, which makes them more accessible for intermolecular interactions.

**Figure 7:**
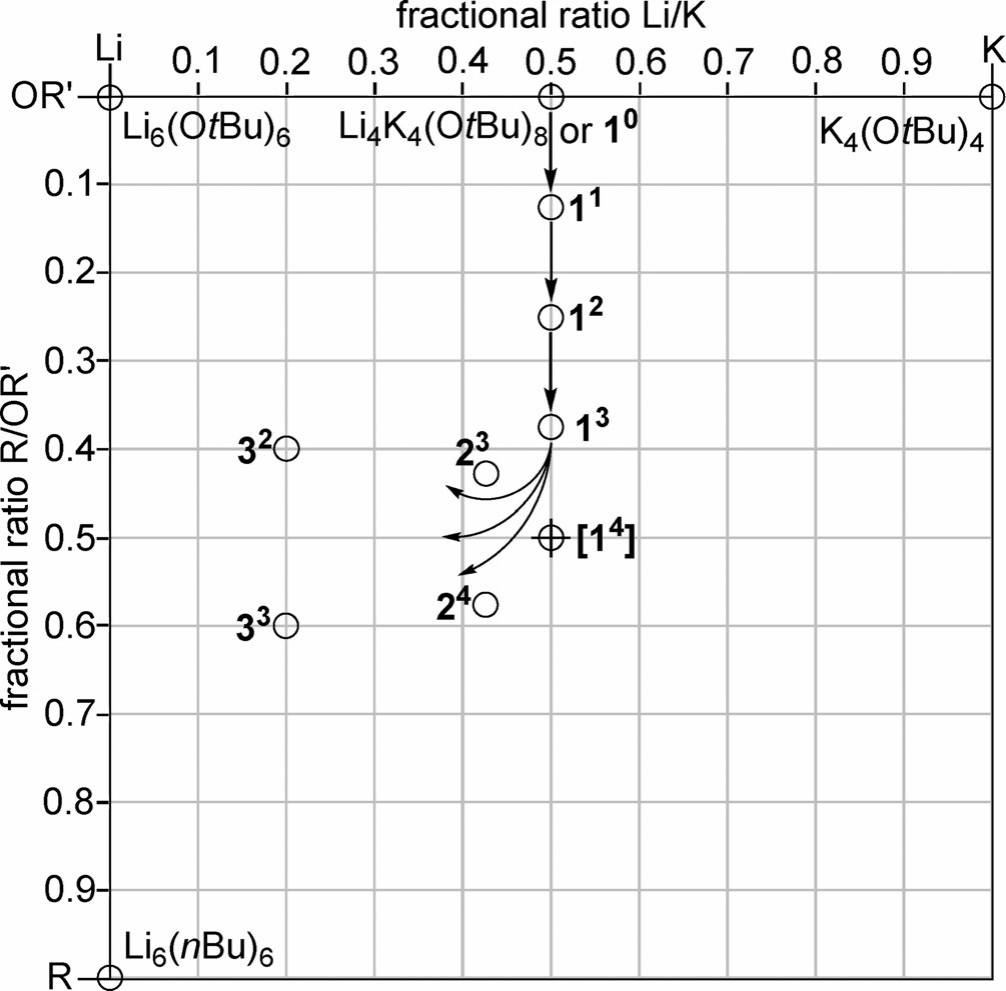
Graphic representation of the successive exchange of *tert*‐butoxy groups by neopentyl groups in Li_4_K_4_(O*t*Bu)_8_ (**1^0^**), leading to the formation of neopentyl‐enriched compounds **1^1^**, **1^2^**, and **1^3^**. Before reaching the ideal 1:1 combination of LiR/KOR’ (**1^4^**), the system evades this composition by producing lithium‐rich compounds such as **2^3^** and **3^2^** (see Scheme 8).

The interplay of compounds **1**, **2**, and **3** can be interpreted graphically (Figure 7): Starting from Mulvey's compound Li_4_K_4_(O*t*Bu)_8_ (**1^0^**,situated on the middle of the top edge of the diagram) it is possible to perform a progressive replacement of O*t*Bu groups with Np. The result is a movement downwards towards the middle of the diagram, passing compounds **1^1^**, **1^2^**, and **1^3^**. Starting with compound **1^3^**, the system is affected by emerging equilibria in solution. This prevents reaching hypothetical LSB Li_4_K_4_Np_4_(O*t*Bu)_4_, **1^4^**, and leads to the formation of compounds enriched with lithium such as compounds **2** and **3** instead. According to their composition, compounds of the family **2** and **3** are found in the left half of the diagram.

While the compounds Li_4_K_4_(O*t*Bu)_8_, **1^1^**, and **1^2^** can be observed in solution with some confidence, compounds **1**, **2**, and **3** were isolated as crystals from such solutions. In solution, the broader ^1^H NMR signals of **2**, **3**, and **1^4^** are indistinguishable or cannot be assigned because of their participation in fast equilibria.^[57]^ However, the absence of strongly coordinating solvents and the structural consistency of solid state and solution in the case of **1**, their presence in non‐donating solvents^[6]^ such as *n*‐hexane or cyclohexane can be anticipated. In reference to the classical LSB, which uses *n*‐butyl groups, it is likely that the stability of an *n*‐butyl compound of a formulation similar to **1**, loses its structural integrity in an even earlier stage. In analogy to **1**, higher *n*‐butyl contents in such compounds would cause ejection of *n*‐butylpotassium units due to its negligible solubility. Still, the presence of a compound such as Li_4_K_4_(*n*Bu)(O*t*Bu)_7_ can be anticipated in low concentrations in solution.

## Structural Motifs, Part 1: Mixed Aggregates

**Scheme 9:**
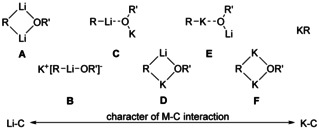
A collection of possible mixed aggregates in mixtures of alkyllithium and potassium alkoxide. In the structural motifs on left side lithium plays the dominant role, on the right side the potassium carbon interactions are more important.

A number of structural motifs potentially present in Lochmann–Schlosser superbases were discussed by Schlosser,^[27]^ which he deduced from the combination of the involved components. These motifs (Scheme 9) range from merely “activated alkyllithium” to pure alkylpotassium. Schlosser suggested the existence of an ate‐complex, a potassium alkyl/alkoxy lithiate (**B**), alkyllithium coordinated to potassium alkoxide (**C**), alkylpotassium coordinated to lithium alkoxide (**E**), and also a mixed aggregate^[37]^ (or symmetrical adduct) of alkyllithium and potassium alkoxide (**D**). A description as “co‐complex”^[37d]^ (of LiR and KOR’) emphasizes the mixed‐metal character of these combinations; the notation “mixed aggregate” also involves homo‐ or uni‐metallic systems.^[31]^

**Figure 8:**
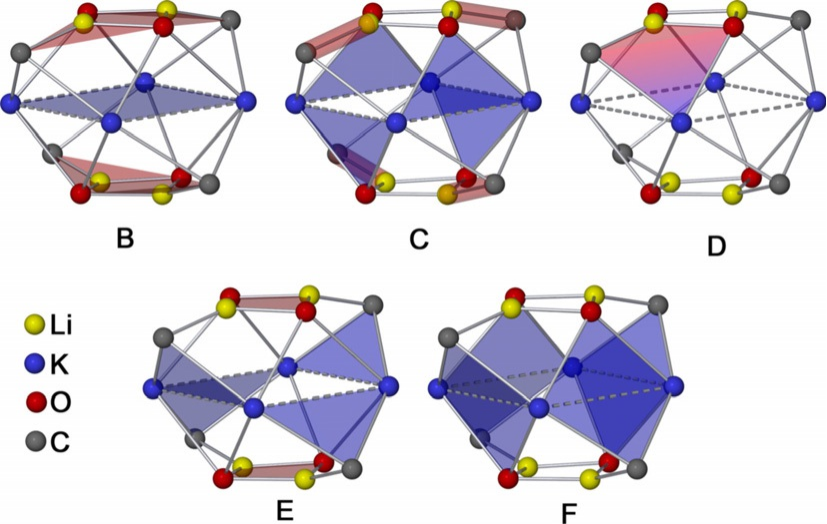
The structural motifs of mixed aggregates shown in Scheme 9 highlighted in the hypothetical structure of **1^4^** derived from compound **1**. Red surfaces illustrate lithium centered molecular units, such as lithiates ([R‐Li‐OR’]_2_, **B**), alkyllithium (Li‐R, **C**), or lithium alkoxide ([LiOR’]_2_, **E**). Blue surfaces represent potassium‐dominated motifs (**B**: K_4_ tetra‐cationic arrangement; **C**: [KOR’]_4_; **E**: [KR]_4_; **F**: [K_2_R(OR’)]). In motif **D**, the red‐blue surface represents one of the eight [LiKR(OR’)] units.

A mixed lithium alkyl/alkoxy compound^[57]^ (**A**) was not considered because of its lower reactivity.^[23]^ With a higher degree of aggregation (hetero‐tetramer or higher aggregated), it is likely to find two or more of these motifs in mixed aggregates; there is also room for interpretation. In the following this is demonstrated on the structure of hypothetical compound **1^4^** derived from structure of **1** (Figure 8).

By regarding the interactions of the four potassium atoms as purely electrostatic, the two remaining units have to be anionic, making the compound a potassium lithiate (**B**). The structure of hypothetical **1^4^** can also be derived from a (open) tetrameric potassium alkoxide,[^41a^, ^42^] which is hosting four monomeric alkyllithium units (**C**). Similarly, the structure of **1^4^** can also be regarded as tetrameric alkylpotassium, which accommodates two dimeric lithium alkoxide units (**E**). The motifs **C** and **E** show the characteristics of both, the starting materials [LiR and KOR’] and the products [KR and LiOR’], at the same time. The same situation was demonstrated by Lochmann on the basis of a hetero‐dimer.^[23]^

The separation into lithium alkoxide units and alkylpotassium can be seen even clearer in Li_4_K_3_Np_4_(O*t*Bu)_3_, **2^4^**, where one potassium atom of a tetrameric K_4_Np_4_ unit (or K_4_Np_3_O*t*Bu unit in **2^3^**) is replaced by a cationic Li_4_(O*t*Bu)_3_ unit. Similarly, dimeric **3^2^** can be considered as a dimeric [KNp]_2_ unit, which is coordinated by two Li_4_Np(O*t*Bu)_3_ units. Motif **D** is present in compounds of the families **1**, **2**, and **3** as a distorted square, where lithium and potassium are bridged by both alkoxide oxygen and an alkyl carbon atom. Another arrangement present in **1^4^** is a square formed by two potassium atoms and the alkoxide oxygen and the alkyl carbon atom (**F**). This motif was not anticipated before. In compound **1^4^**, it would be consistent with a chemically less meaningful lithium potassiate.^[58]^ However, in a mixed alkyl/alkoxy potassium compound (in the absence of lithium) it would be the only relevant motif.

## Degradation of Mixed Aggregates Following Metalation Reactions

**Scheme 10:**
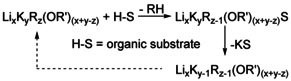
Reaction of a mixed aggregate with an organic substrate S‐H leading to the formation and elimination of a potassiated substrate. The reaction leads to a formal loss of alkylpotassium from the mixed aggregate. The residual mixed aggregate can act as a base itself again or take part in equilibria, forming new basic species enriched with lithium and alkoxide.

The transformation of hypothetical **1^4^** into **2^3^** by loss of one unit of neopentylpotassium (Scheme 8) provides an insight into what happens to the base, when it is consumed in a reaction with an acidic substrate. A proton is transferred from the acidic substrate to an alkyl group, which is then released to the solution as alkane. The deprotonated substrate anion replaces the alkyl group in the mixed aggregate (Scheme 10).

**Figure 9:**
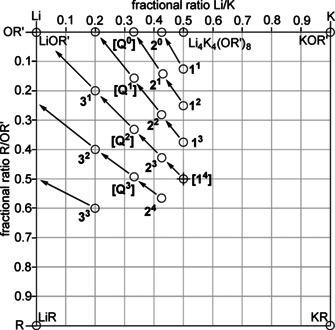
Graphical representation of reaction sequences during the reaction of mixed aggregates with an organic substrate. The arrows represent the formal loss of an alkylpotassium unit, leading to a new mixed aggregate. **[Q^z^]** represents a hypothetical hetero‐hexamer Li_4_K_2_R_*z*_(OR’)_(6−*z*)_. The final product will be LiOR’, or considerably less basic mixed aggregates of the formula Li_*x*_K_*y*_(OR’)_(*x*+*y*)_ or Li_*x*_R_z_(OR’)_(*x*−*z*)_, depending on the original composition.

In contrast to the “monodentate” alkyl group with a single directed interaction, the anion of the substrate anion (e.g. a phenyl anion) will exhibit additional electron pairs allowing secondary (intermolecular) interactions to potassium cations.^[2b]^ This will result in the precipitation of the insoluble corresponding potassium‐substrate compound, leaving behind a mixed aggregate lacking one alkylpotassium unit. The consequence is a new compound enriched by both lithium and alkoxide, resulting in a movement to the upper left corner away from KR in the mixed aggregate diagram (Figure 9). This reaction path can be conceived for every known or hypothetical mixed aggregate containing highly basic alkyl groups. A possible, but not yet observed, mixed aggregate in the sequence Li_4_K_4_Np_z_(O*t*Bu)_(8−*z*)_ (*z=*3: **1^3^**) and Li_4_K_3_Np_z_(O*t*Bu)_(7−*z*)_ (*z=*3: **2^3^**) would be hetero‐hexameric Li_4_K_2_Np_z_(O*t*Bu)_(6−*z*)_ (**Q^Z^**). This hypothetical compound **Q** of unknown structure would lead, after formal loss of another KNp unit, to Li_4_KNp_z_(O*t*Bu)_(5−*z*)_ (*z=*2: **3^2^**). After a final step, LiO*t*Bu will stay behind, which in turn can interfere with all the other neopentyl‐containing species to form lithium alkoxide enriched species.

In Figure 9, the graphical representation of a reaction sequence (e.g. **1^4^**, **2^3^**, **Q^2^**, **3^1^**, LiO*t*Bu) follows a line, with KNp (the lower right corner) as radiant. Hence, each formal loss of KNp is connected to a vector/arrow pointing away from KNp.

## Homometallic Potassium Mixed Aggregates

The successive transformation of one mixed aggregate into another during a reaction with an organic substrate and the participation of these compounds in interchanging equilibria is a major obstacle when it comes to the description of the reactivity of the involved bases. The assignment of both NMR and vibrational spectroscopic data of involved chemical groups to distinguishable species will not be an easy task. Isotopically enriched compounds may help to decipher such systems.

**Figure 10:**
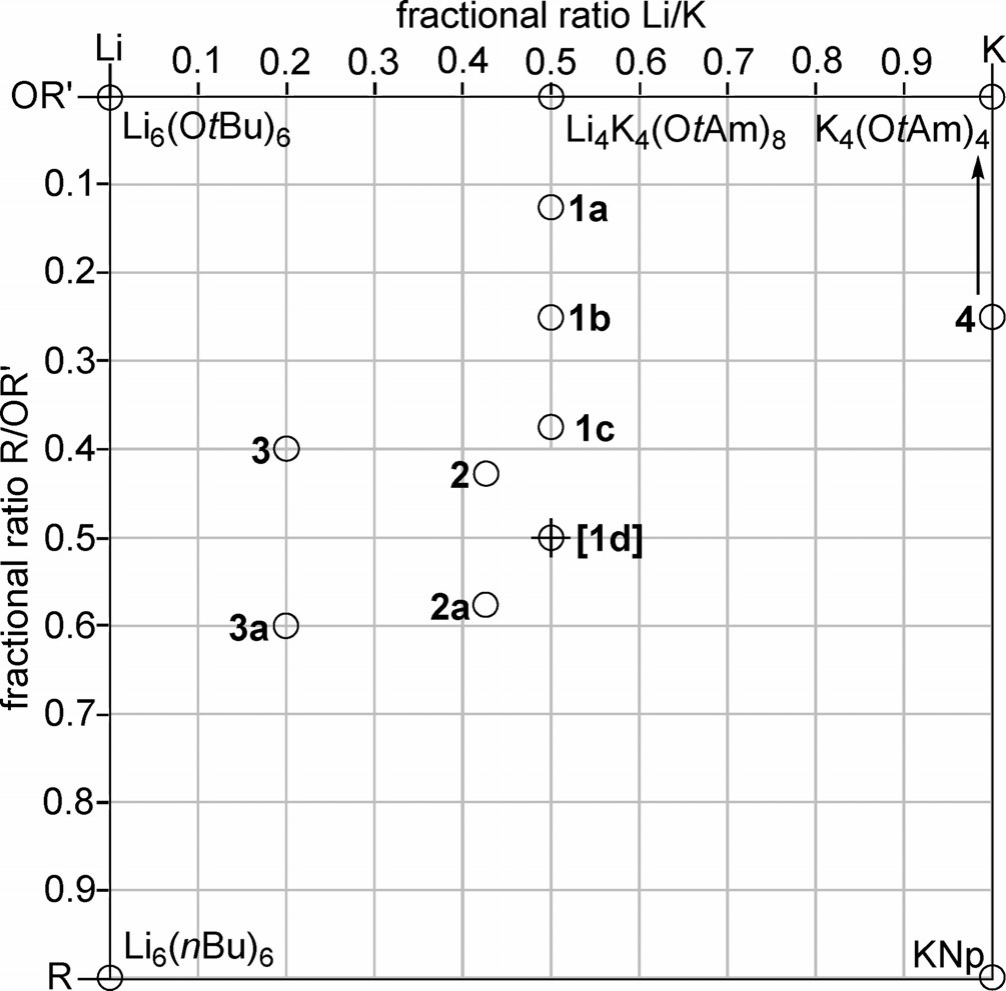
Graphic representation of the chemical composition of compound **4**, respectively **4^1^**. The arrow represents the formal loss of a neopentylpotassium unit during a reaction with an organic substrate, producing potassium alkoxide.

The formation of mixed aggregates without lithium will simplify matters considerably. Leaving out lithium as a fourth component, it is possible to find out whether the cooperativity (or synergy) of two different metals^[59]^ or the presence of both alkyl and alkoxy groups side by side is required to obtain superbasicity. The synergy of numerous mixed‐metal systems usually depends on the reactivity‐enhancing effect of a polar metal compound on a second less reactive, less polar organometallic compound, while the reactivity of bi‐ and homo‐metallic LSBs or related mixed aggregates is described by the taming effect of the added alkoxide on the fiercely reactive alkali metal alkyl compound. The potential of homometallic bases was demonstrated by the outstanding reactivity of sodium alkyl/alkoxy mixtures^[25]^ or even by a lithium amide/alkyl mixture, which was able to metalate cyclopentadienyllithium for a second time.^[60]^ Bases consisting of potassium alkoxide and alkylpotassium can be expected to show less structural diversity and even higher basic reactivity compared to systems using lithium and potassium. In the diagram representation of mixed aggregates, these potassium compounds are found only on the right edge of the diagram (Figure 10.)

**Scheme 11:**
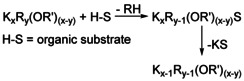
Reaction of a potassium mixed aggregate with an organic substrate S‐H leading to the formation of a potassiated substrate. The reaction leads to a formal elimination of alkylpotassium from the mixed aggregate, the residual compound is inactive as base in this context if *y=*1.

The same is true for products based on a reaction of this base with organic substrates and subsequent removal of insoluble potassium product compound (Scheme 11). The residual mixed aggregates will contain more alkoxide in relation to alkyl groups. Ultimately, a solution of potassium alkoxide will be left, which can easily be separated from the insoluble potassium compound. The latter compound could also contain stoichiometric amounts of alkoxide in some cases.^[61]^

**Scheme 12:**
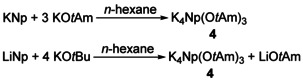
Formation of compound **4** by reaction of neopentylpotassium with three equivalents potassium *tert*‐amyloxide or by reaction of neopentyllithium (LiNp) with excess potassium *tert‐*amyloxide (KO*t*Am).

The formation of a potassium mixed alkyl/alkoxy aggregate was achieved by dissolving KNp in a solution of potassium *tert*‐amyloxide (potassium 2‐methyl‐2‐butoxide, KO*t*Am).^[54]^ The latter compound was used because of its increased solubility in non‐donating solvents such as *n*‐hexane.^[42]^ However, the poor thermal stability of KNp made it favorable to use LiNp with an excess of KO*t*Am (Scheme 12). This produces KNp in situ, the by‐product LiO*t*Am is trapped simultaneously by the excess KO*t*Am to form Li_4_K_4_(O*t*Am)_8_. Interestingly, no formation of an aggregate was observed by mixing *n*‐butylpotassium and KO*t*Am.^[61a]^

**Figure 11:**
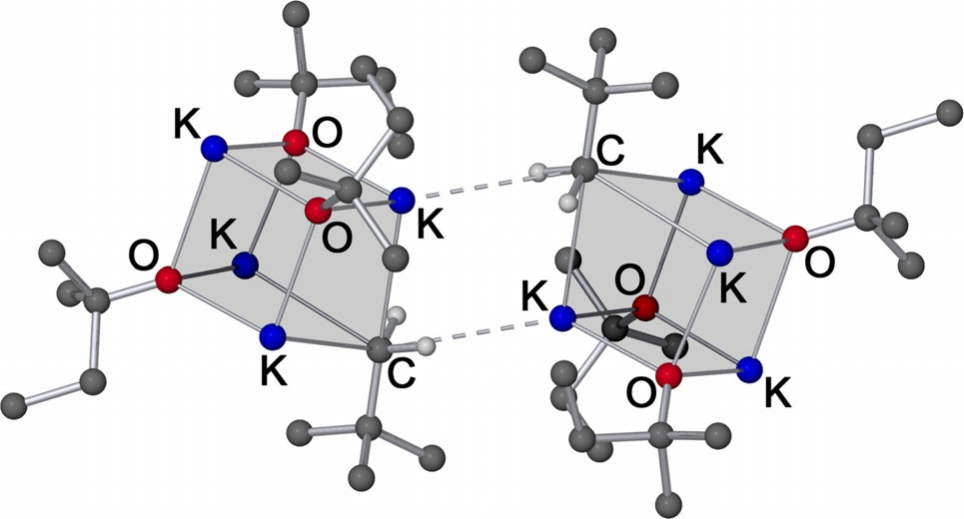
Molecular structure of dimeric compound **4**. Minor occupied disordered units and hydrogen atoms of the alkoxy and neopentyl groups are omitted for clarity. K_4_O_4_‐cubanes are shaded for emphasis.

The product, which was isolated as large yellowish crystals from a solution of *n*‐hexane at −30 °C, was identified as K_4_Np(O*t*Am)_3_ (**4**) by NMR spectroscopy and X‐ray diffractometry. The compound consists of a hetero‐tetramer of one KNp unit and three KO*t*Am units arranged in a hetero‐cubus (Figure 11).

In contrast to pure potassium alkoxides, which preferably crystallize in tetrameric form,[^41a^, ^42^] the mixed aggregate **4** (=**4^1^**) offers an additional (nucleophilic) coordination site for the coordinatively unsaturated potassium atoms. The interaction is bilateral, which results in the formation of a dimer of hetero‐tetramers (similar to compound **3**). This interaction is easily released in solution, as could be demonstrated by DOSY NMR^[62]^ of **4** in deuterated cyclohexane. A decrease in concentration resulted in the formation of a lighter, more mobile neopentyl‐containing species. The formation of a hexameric form cannot be ruled out, but the preference of potassium alkoxide[^41a^, ^42^] and alkylpotassium[^13^, ^51^] for the formation of tetramers makes this unlikely. The example of this dimerization might also hint to what happens to more neopentyl‐rich hetero‐tetramers. K_4_Np_2_(O*t*Am)_2_ (**4^2^**) can be expected to form badly soluble linear or zig‐zag polymeric chains, similar to the structure of [NaCH_2_SiMe_3_]_∞_.^[9c]^ Accordingly, the likewise hypothetical forms K_4_Np_3_(O*t*Am) (**4^3^**) and tetrameric KNp (by analogy: **4^4^**) might form two‐ or three‐dimensional networks in the same way.

In contrast to pure KNp,^[50]^ compound **4** has a higher thermal stability, demonstrated by a slow decomposition over several hours as solid or in solution. This adds a stabilizing effect to the solubilizing capabilities of excess potassium alkoxide.

In addition, the comparable simple arrangement of **4** exhibits motif **F** (Scheme 9), a four‐membered ring of two potassium atoms bridged by an alkyl group and an alkoxy group, respectively. In this instance, it is comparable to compounds **1** and **2**; however, because of the absence of lithium, all the other motifs (**A**‐**E**) are excluded in **4**. Overall, the presence of compounds similar to **4** can be expected in superbasic mixtures using an excess of soluble potassium alkoxides.^[61]^

## Structural Motifs, Part 2: Alkyl–Metal Interactions

**Scheme 13:**
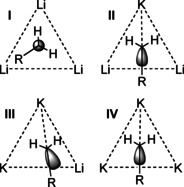
Possible homometallic and heterometallic environments for alkali metal/alkyl interactions in mixed lithium/potassium compounds. The tri‐metallic platform includes the motifs Li_3_ (**I**), Li_2_K (**II**), LiK_2_ (**III**), and K_3_ (**IV**). The dashed lines represent topological metal‐metal distances and agostic type potassium/CH_2_‐interactions.

Taking into account the presence of several motifs (Scheme 9 A **–F**) in a single compound and the possible coexistence of several species in solution, it is very difficult to connect these structural features to the reactivity of such mixtures. The focus on the environment of the very basic alkyl group leads to a less complicated picture. In this approach, the role of the alkoxy groups is more or less reduced to the role of a chemically inert structural support or solubilizing co‐reagent. In many alkyl lithium compounds and in the neopentyl mixed aggregates **1**, **2**, and **3**, the alkyl group is found in a μ_3_ bridging position between three metal atoms. A μ_4_ position was found only for the oxygen atom of alkoxy groups. This results in four possible environments for the metalated α‐carbon atom: Li_3_ (**I**), Li_2_K (**II**), LiK_2_ (**III**), and K_3_ (**IV**) (Scheme 13).

Motif **I** is found in numerous alkyl compounds of lithium.^[3]^ Motifs **II**, **III**, and **IV** are very rare in structurally characterized alkyl potassium compounds. With the exception of ill‐defined methyl sodium with lithium atoms statistically replacing sodium atoms,^[13]^ there is no information available about mixed alkali metal alkyl compounds. While there are numerous examples of combinations of alkali metals with less electropositive metals such as magnesium or zinc,^[59]^ the structural motif of alkyl groups coordinated by a mixed alkali metal environment is so far restricted to compounds **1** (motif **III**),^[52]^ **2** (motifs **III**+**IV**),^[52]^ and **3** (motif **II**)^[54]^ with their mixed alkyl/alkoxy arrangement. Motif **IV** with a K_3_ environment is also present in pure alkyl potassium compounds such as polymeric methyl potassium^[63]^ or tetrameric trimethylsilylmethyl potassium coordinated by TMEDA.^[51]^

**Figure 12:**
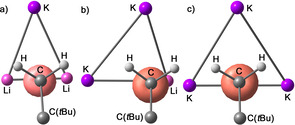
Result of theoretical investigations showing the coordination of the neopentyl group to a tri‐metallic platform in **3** (a, Li_2_K, motif **II**), **1** (b, LiK_2_, motif **III**), and **2** (c, K_3_, motif **IV**). Only the relevant atoms are shown, the view is in direction of the free electron pair (red orbital) of the CH_2_ α‐carbon atom (level of calculation: B3LYP/6‐31G*).

Due to the small number of available relevant structures and the positional disorder of the involved neopentyl groups, it is difficult to obtain a reliable picture of the steric and electronic bonding situation between an alkyl group and a mixed metallic environment. However, in the case of motif **III** and **IV**, the findings are backed by computational models of compounds **1** and **2**.^[52]^ The metalated α‐carbon of a primary alkyl group is bonded to a trimetallic lithium platform via a four‐center two‐electron bond,^[64]^ if an substantial covalent contribution to the interaction is assumed. Based on the positions of the attached alkyl group and the (less reliable) positions of the hydrogen atoms, it is possible to speculate about the position of the electron pair of the sp^3^ hybridized carbon interacting with the metal atoms. In case of alkyllithium compounds, the electron pair points towards the space between the three lithium atoms. Here, the orientation of the attached alkyl group and the two hydrogen atoms also depends on steric interactions such as β‐CH_2_ lithium attractions. In some basic lithium compounds, this leads to an eclipsed conformation^[55]^ according to the three lithium atoms (motif **I**, Scheme 13). If one or more potassium atoms are present in the trimetallic platform, a different structural motif appears: the potassium atom, the α‐carbon, and the carbon of the alkyl group (here: neopentyl) form an angle larger than ≈160°. This approximately linear formation places the two protons of the neopentyl‐CH_2_ unit in close proximity of the potassium atom, comparable to an agostic interaction. In motif **II**, which is present in the (barely disordered) structure of compound **3**, the position of the *tert*‐butyl group (as well as the two protons) suggests that the electron pair of the carbon‐metal interaction points in the middle of the two lithium atoms (Figure 12). This would be in accordance with a more covalent three‐center two‐electron bond between carbon and lithium and a more electrostatic interaction between carbon and potassium. Motif **III** also exhibits the linear K‐alkyl arrangement and a direct interaction of lithium with the α‐carbon atom. However, structural and computational data suggest that the electron pair of this interaction is slightly displaced towards the second potassium atom (Figure 12).

Similar to the tri‐lithium motif **I**, the α‐carbon atom is situated rather symmetrically over the K_3_ triangle in motif **IV**. However, the linear K‐C_α_‐C arrangement causes the “free” electron pair at the α‐carbon pair to point towards the area between the two other potassium atoms.

Regarding the structures of compounds **1**, **2**, and **3** and the intrinsic structural motifs, it is an interesting point that the α‐carbon atom of the neopentyl group is always situated over a trimetallic platform possessing as many potassium atoms as possible. However, the explanation for this behavior lies not in a conceivable affinity of the alkyl group towards potassium. The ability of lithium to form (polar) covalent bonds to carbon in comparison to more ionic or electrostatic potassium carbon interactions (Li‐C: covalent but polar; K‐C: mainly electrostatic; see Scheme 13, Figure 12) also contradicts this purely topological fact. The reason is found in the optimal interaction between lithium as hard Lewis acid and alkoxide as hard Lewis base, forcing potassium atoms and alkyl groups into the same structural corner. The structural O*t*Bu/Np mimicry prevents the expulsion and self‐aggregation of the soft Lewis acid/base pair KNp from the complex and ending up in an insoluble compound. Accordingly, the Np group is tolerated in the soluble mixed aggregate, although finding itself in a sterically exposed situation promoting its reactivity. The same situation is conceivable for other alkyl groups, although in lower concentration or in solid state. In this enforced mismatch of potassium and alkyl group, which results in a tempestuous chemical relationship, lays an important reason for the singular reactivity of these mixed aggregates.

## Structural Motifs and Superbasicity

The essential part of every organometallic superbase is an alkyl group bonded to one or more electropositive metals. In the case of mixed aggregate alkali metal superbases, this central alkyl/alkali metal arrangement is supported by further alkali metal alkoxide units. A direct involvement of the alkoxy groups as intermediate base cannot be ruled out so far. Similar two‐step reactions were observed in mixed metal alkyl/amide bases,^[65]^ but here, the basicity of the involved amido groups is considerably higher than the basicity of *tert*‐alkoxides.^[66]^ It is much more plausible that the alkyl group alone is acting as basic group in corresponding transition states.

Considering the vital role of the alkyl group, the bonded alkali metal atoms will have a more dominant effect on its reactivity than their coordination by alkoxide anions. Then the main tasks of the alkoxides would be to provide the architecture for the tri‐metallic platform supporting the alkyl group, and to enhance the stability and solubility of such aggregates.

**Scheme 14:**
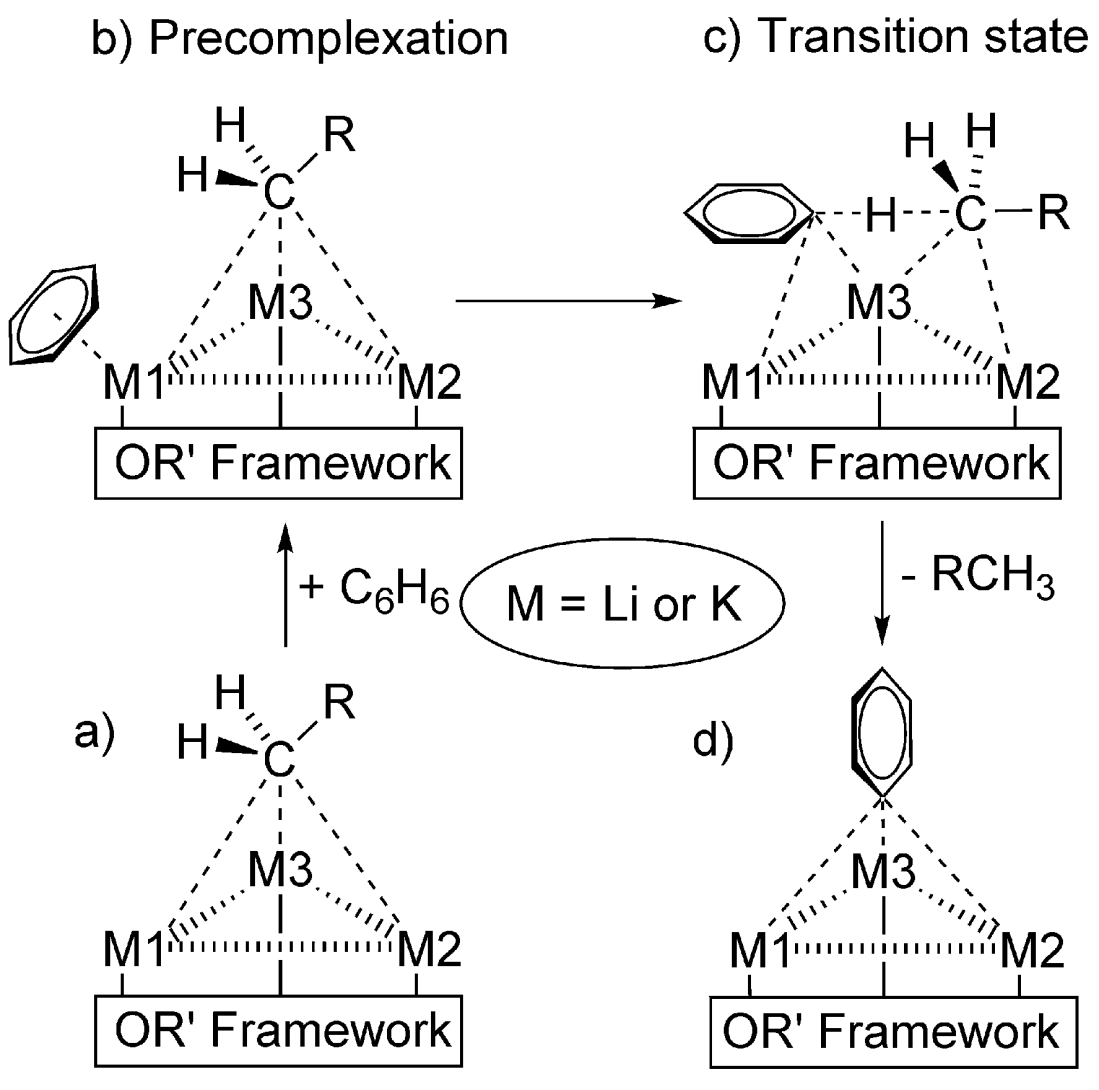
Hypothetical path of a reaction of a mixed aggregate with benzene. Only interactions of the alkyl group, benzene, and the phenyl group with the metals of the tri‐metallic platform are provided. M1, M2, and M3 are lithium or potassium, respectively. The steps shown involve the mixed aggregate (a), the coordination of benzene to one of the alkali metals (b), the transition state (c), and the new phenyl compound (d).

The constitution of the tri‐metallic platform will have considerable influence of the reactivity of the alkyl group. First, the electro‐positivity and the size of the involved alkali metals will have a large effect on the metal carbon bond polarity. In other words, the stabilization of the negative charge of the carbanionic alkyl group will also affect the basicity. Second, the Lewis acidic nature of the alkali metal (Li: hard, K: soft) offers organic substrates a docking site in advance of metalation.^[67]^ Functional groups in the substrate with donor atoms such as nitrogen or oxygen will prefer lithium as ‘Lewis acid, while softer π‐electron system of aromatic compounds will interact preferably with potassium atoms.^[68]^ Mixed metal species can offer both coordination modes in the same time. In the course of a metalation, it is also important to consider the reaction path including the transition state and the formed products (Scheme 14). The structural motifs **I**–**IV** will have a considerable influence of the energy of the transition state, which will determine the kinetics of the reaction and therefore the regio‐selectivity of the outcome. The energetic stabilization of the final products is also of similar importance.

It is obvious that each organic substrate will show a different behavior towards the metalation platform, depending on the number of involved lithium and potassium atoms, respectively. This applies to the pre‐complexation, the transition state, and the ultimate product.

An actual study of reactions, which will shed light on the reactivity of these structural motifs, will be complicated by a number of problems. These include the co‐existence of different structural motifs in the same molecule, interchanging equilibria between different species, and the “morphological evolution” during the reaction. Theoretical calculations, which allow the study of well‐defined species, are an alternative.

## Assessment of the Actual Basicity of Alkali Metal Superbases

The practical determination of the absolute basicity of superbasic alkali metal compounds is not an easy task, because of their high reactivity and poor solubility. This is further complicated by the coexistence of different species with several potentially basic sites and ongoing interchanging equilibria in solution. The poor solubility of the products formed in the course of a metalation makes it impossible in most cases to study the position of the equilibrium (thermodynamic basicity) or the speed of the reaction (kinetic basicity).^[7a]^ Another possibility is to find the metalation threshold^[35]^ of such bases by checking which hydrocarbon can be metalated and which not.^[15]^ However, if only aliphatic or cyclic hydrocarbons such as pentane or cyclohexane escape metalation by Lochmann–Schlosser bases, then there is little space left for further differentiation of the basicity of such bases.

One characteristic, which is restricted to alkali metal superbases, is the possibility of polymetalations of arenes. Dimetalations of benzene^[69]^ and naphthalene[^69b^, ^70^] were achieved exclusively by bases containing the heavier alkali metals sodium or potassium.

Several poly‐metalations of ferrocene were achieved by compounds including heavier alkali metals,^[71]^ even tetra‐metalations by mixed metal (synergic) sodium magnesiates and very recently also by sodium zincates are reported.^[72]^ But also alkyllithium or donor‐activated alkyllithium are able to perform polymetalations.^[73]^

## Metalation of Ferrocene with Potassium Alkyl/Alkoxy Aggregates

**Scheme 15:**
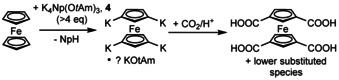
Reaction of excess K_4_Np(O*t*Am)_3_, **4** with ferrocene and subsequent reaction with CO_2_ and acidic work‐up.

Compound **4** was successfully used in a tetra‐metalation of ferrocene.^[54]^ Ferrocene is a suitable test‐substrate for metalation for several reasons. It is air‐stable, solid and can easily be added in small quantities; the yellow‐orange color of ferrocene changes to red when metalation occurs; finally, metalated ferrocene is reasonably stable at ambient temperature and can be reacted with a range of electrophilic reagents. However, like many other examples of poly‐metalated ferrocene, the red metalated product of the reaction of ferrocene with a five‐fold excess of **4** formed in situ in *n*‐hexane is completely insoluble in inert solvents and highly reactive. This prevented the spectroscopic and structural characterization of the metalated solid. Despite the solubility of **4** in *n*‐hexane, the product of this reaction shares the same fate as countless other aromatic compounds metalated by Lochmann–Schlosser superbases: their composition and structures remain a mystery. A destructive hydrolysis in this case proved the presence of ferrocene and alkoxide, and absence of neopentane in the metalated product. The reaction with excess CO_2_ led to the formation of 1,1’,3,3’‐ferrocenetetracarboxylic acid in yields close to 80 % besides smaller amounts of di‐ and tri‐substituted ferrocenes (Scheme 15).

It is reasonable to assume that the observed yields of the CO_2_‐trapped product represent the metalated species; there are no hints towards substantial hydrolysis on one side or post‐quench metalation processes on the other. In this respect, compound **4** demonstrated a more efficient tetra‐metalation of ferrocene in comparison to other examples using Lochmann–Schlosser superbases. This outstanding reactivity is not restricted to neopentyl compounds. *n‐*Butyllithium with an excess of KO*t*Am used under the same conditions also achieves the tetra‐functionalization of ferrocene with yields close to 60 %.^[74]^

## Conclusions from Metalation Reactions Using Potassium Mixed Aggregates

Assuming a similar basicity of *n*‐butyl and neopentyl,^[75]^ the difference in the respective yields in these otherwise similar reactions arises from changes in solubility. When *n*‐butyllithium is added to a large quantity/amount of KO*t*Am,^[54]^ the absence of an immediate precipitation suggests the formation of a mixed aggregate such as K_4_(*n*Bu)(O*t*Am)_3_. This finding is in contrast to a report by Lochmann,^[61]^ where the reaction was achieved conversely.

In addition, the successful tetra‐metalation of ferrocene by compound **4** demonstrates that homo‐metallic potassium aggregates are equal to other superbases in terms of basicity. In this case, the reactivity can be connected directly to structural motifs **F** (Scheme 9) and **IV** (Scheme 13). There was no substantial difference in the obtained yield of substituted ferrocene, when **4** was produced in situ by mixing LiNp with KO*t*Am or by mixing KNp with three equivalents of KO*t*Am. In the former case, this indicates that lithium is trapped in inactive compounds such as Li_4_K_4_(O*t*Am)_8_ (**1^0^**) or Li_4_K(O*t*Am)_5_ (**3^0^**).^[47]^

**Scheme 16:**
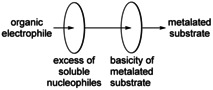
Obstacles of a reaction of an organic substrate metalated by Lochmann–Schlosser superbases with an organic electrophile.

The reaction of metalated species produced by Lochmann–Schlosser superbases with electrophiles involves some obstacles (Scheme 16). The metalated species are more or less insoluble, which does not significantly affect the reaction if the electrophile is reactive enough and/or soluble itself. Of more importance is the fact that the alkali metal alkoxide, which is present in excess and in higher concentrations, also acts as a nucleophile. This is no problem when the electrophile, such as CO_2_ or I_2_, can be used in excess, or if the metalate species can be separated from excess alkoxide by filtration. Finally, the metalated species itself is a potassium compound with a substantial basicity. Electrophiles with acidic hydrogen atoms (such as benzylic or allylic groups) are at risk to be metalated themselves before a successful functionalization of the metalated carbon atom can be achieved. Metal–metal exchange reactions^[76]^ (such as potassium‐zinc exchange), which could lift the restriction to proton‐free electrophiles, introduce new synthetic problems.

To conclude, relevant pieces of information can be drawn from the first metalation reactions using compound **4**. First, mixed alkali metal mixed aggregates do not lose their singular reactivity in the absence of lithium. A possible mixed metal synergy is therefore not essential. Second, the difference in solubility in mixtures using neopentyl or *n*‐butyl groups reflect itself in the yields of the final product, but not in overall outcome such as changing degrees of polymetalation or regioselectivity. The main features of the neopentyl group in reference to the *n*‐butyl group are the absence of missing metal‐β‐CH_2_ interactions, which is also causing the inability of β‐elimination, and the “mimicry” of *tert*‐butoxy groups. Due to its greater stability and solubility, this makes the neopentyl group a feasible test case for the much more popular and commercially available *n*‐butyl compounds used in Lochmann–Schlosser superbases.

## Summary and Outlook

For many years after their discovery, the composition of Lochmann–Schlosser superbases could not be determined conclusively based on mixtures of alkyllithium and potassium alkoxides. The use of the neopentyl group in such mixtures leads to products soluble in *n*‐hexane or other alkanes. This way, it was possible to perform NMR studies in solution and to obtain crystals for structural studies. The solid‐state structures of these compounds simultaneously revealed genuine mixed aggregates containing lithium, potassium, alkyl, and alkoxy groups. Depending on the amount of initial materials, the ratio of the components varied, the mixed aggregates showed an excess of lithium and/or alkoxy groups. In solution, it is possible to identify alkoxy‐rich Li_4_K_4_‐hetero‐octamer by NMR spectroscopy. Increasing the alkyl content leads to the equilibrium of lithium‐richer aggregates, which are undistinguishable by NMR spectroscopy. When alkyllithium is combined with an excess of potassium alkoxide it is possible to isolate a potassium alkyl/alkoxy aggregate. The basicity of this compound could be demonstrated by a synthetically useful tetra‐metalation of ferrocene. A number of structural motifs were identified in structurally known mixed aggregates. These motifs can be derived from the involved starting materials and products or from connectivity of the alkyl groups to the alkali metals. Superbasicity of mixed aggregates can be observed in the absence of lithium, but the presence of potassium or other heavier alkali metals is mandatory. Alkali metal alkoxide provide a solubilizing framework for otherwise insoluble and rather unstable alkylpotassium, which has a positive effect on the reactivity of such aggregates. One has to bear in mind, that alkyl alkali metal compounds represent Lewis acid/base complexes, where the Lewis acidic needs of the alkali metal atoms are hardly met by the Lewis basicity of the alkyl groups. Addition of alkali metal alkoxide introduces new Lewis basic groups but also Lewis acidic metal atoms in the same time. This results in a predominantly Lewis acidic behavior, which makes the mixed aggregates susceptible for all kinds of Lewis basic molecules, even more so in non‐donating solvents such as *n*‐hexane.

In consideration of the fact that all reactions with alkali metal superbases are carried out in solution, it is important to gain more information about the behavior of these compounds in the solution phase. By introduction of NMR‐active isotopes, such as ^6^Li, ^2^H, ^133^Cs, or ^13^C, and the use of sophisticated DOSY NMR techniques, it will be possible to identify the present species spectroscopically and to study the kinetic and thermodynamic properties by NMR spectroscopy. Additional solid‐state structures of relevant alkali metal compounds will fill important gaps. The existence of alkyl/alkoxy mixed aggregates other than compounds **1**, **2**, **3**, and **4** is quite feasible. This would include similar sodium, rubidium, and cesium compounds. Still unknown structures of alkylpotassium or alkylsodium compounds, but also structures of mixed alkali metal alkyl compounds, such as Li_*x*_K_*y*_R_*z*_, are of great interest. Furthermore, understanding the reactivity of alkali metal superbases would benefit substantially if more could be learned about the nature of the metalated substrates. New synthetic strategies may lead to soluble products, allowing their characterization. In the same time, it could be possible to perform trans‐metalation reactions, opening up new synthetic routes with a wide range of organic nucleophiles enabling cross‐coupling reactions.

## Conflict of interest

The authors declare no conflict of interest.

## Biographical Information

*Jan Klett studied chemistry at the University of Stuttgart. He finished his doctoral thesis 2006 in Mainz. In 2006, he worked as a postdoctoral researcher with Robert E. Mulvey at the University of Strathclyde, followed by a fellowship of the Royal Society of Edinburgh/BP Trust (2009–2012). Currently, he is a junior research group leader at the University of Mainz. In 2017, he received the Arfvedson–Schlenk award*.